# Ovarian cancer variant rs2072590 is associated with HOXD1 and HOXD3 gene expression

**DOI:** 10.18632/oncotarget.21902

**Published:** 2017-10-13

**Authors:** Liyuan Guo, Yan Peng, Lei Sun, Xia Han, Juan Xu, Dongwei Mao

**Affiliations:** ^1^ Department of Gynecological Oncology, Cancer Hospital of Harbin Medical University, Harbin, China; ^2^ Disease Prevention Center, First Affiliated Hospital, Heilongjiang University of Chinese Medicine, Harbin, China; ^3^ Department of Gynecology and Obstetrics, The Fourth Hospital of Harbin Medical University, Harbin, China; ^4^ Shenzhen Hospital of Guangzhou University of Chinese Medicine, Shenzhen, China

**Keywords:** ovarian cancer, genome-wide association study, gene expression

## Abstract

Ovarian cancer (OC) is a common cancer in women and the leading cause of deaths from gynaecological malignancies in the world. In addition to the candidate gene approach to identify OC susceptibility genes, the genome-wide association study (GWAS) methods have reported new variants that are associated with OC risk. The minor allele of rs2072590 at 2q31 was associated with an increased OC risk, and was primarily significant for serous subtype. The OC risk-associated SNP rs2072590 lies in non-coding DNA downstream of *HOXD3* and upstream of *HOXD1*, and it tags SNPs in the *HOXD3* 3′ UTR. We think that the non-coding rs2072590 variant may contribute to OC susceptibility by regulating the gene expression of HOXD1 and HOXD3. In order to investigate this association, we performed a bioinformatics analysis by a functional annotation of rs2072590 variant using RegulomeDB (version 1.1), HaploReg (version 4.1), and PhenoScanner (version 1.1). Using HaploReg, we identified 19 genetic variants tagged by rs2072590 variant with with r^2^ >= 0.8. Using RegulomeDB, we identified that three genetic variants are likely to affect TF binding + any motif + DNase Footprint + DNase peak. Other genetic variants are likely to affect TF binding + DNase peak. Using PhenoScanner (version 1.1), we identified that these 19 genetic variants could significantly regulate the expression of nearby genes, especially the HOXD1 and HOXD3 in human ovary tissue.

## INTRODUCTION

Ovarian cancer (OC) is a common cancer in women and the leading cause of deaths from gynaecological malignancies in the world [1]. Like other human complex diseases, OC is caused by the combination of genetic variants and environmental factors, including the familial BRCA1 and BRCA2 mutations and common genetic variants of lower penetrance [1]. In addition to the candidate gene approach to identify OC susceptibility genes, the genome-wide association study (GWAS) methods have also reported new variants that are associated with OC risk [1].

However, the exact genetic mechanisms for these OC susceptibility variants are still unclear [2]. It is reported that the potential associations between gene expression and OC risk alleles may connect risk variants to their putative target genes/transcripts and biological pathways [2]. The minor allele of rs2072590 at 2q31 was associated with an increased OC risk (OR = 1.16, 95% CI 1.12–1.21, *p* = 4.5 × 10^−14^), and was primarily significant for serous subtype (OR = 1.20, 95% CI 1.14–1.25, *p* = 3.8×10^−14^) [3]. The 2q31 locus contains a family of homeobox (HOX) genes involved in regulating embryogenesis and organogenesis [3]. Altered expression of HOX genes has been reported in many cancers [3]. The OC risk-associated SNP rs2072590 lies in non-coding DNA downstream of *HOXD3* and upstream of *HOXD1*, and it tags SNPs in the *HOXD3* 3′ UTR [3].

We think that the non-coding rs2072590 variant may contribute to OC susceptibility by regulating the gene expression of *HOXD1 and HOXD3.* In order to investigate this association, we conducted a functional annotation of rs2072590 variant using RegulomeDB (version 1.1) [4], HaploReg (version 4.1) [5], and PhenoScanner (version 1.1) [6].

## RESULTS

### LD analysis using HaploReg

Using the LD information from the 1000 Genomes Project (*EUR*), we got 19 genetic variants tagged by rs2072590 variant with with r^2^ >= 0.8. These 19 genetic variants are located around the HOXD4, HOXD3, AC009336.24 and HOXD-AS1. Here, we give the detailed information including the LD information about these variants in Table 1.

**Table 1:** rs2072590 and variants with r^2^ > = 0.8

SNP	chromosome	pos (hg38)	LD (r^2^)	LD (D’)	Ref	Alt	Gene	Functional annotation
rs4972504	2	176153998	0.89	0.98	T	C	HOXD4	
rs2551802	2	176157430	0.85	0.93	C	G	HOXD3	
rs2252895	2	176159192	0.96	0.98	A	G	HOXD3	
rs2252894	2	176159194	0.88	0.96	G	C	HOXD3	
rs2857538	2	176159533	0.98	1	C	T	HOXD3	
rs2857540	2	176161970	0.98	0.99	G	T	HOXD3	
rs2113559	2	176166371	0.97	0.99	A	G	HOXD3	intronic
rs717852	2	176166895	0.98	1	C	T	HOXD3	intronic
rs2249131	2	176167367	0.98	1	C	T	HOXD3	intronic
rs2857532	2	176168555	0.98	1	A	G	HOXD3	intronic
rs1051929	2	176172026	1	1	T	C	HOXD3	synonymous
rs711830	2	176172583	1	1	A	G	HOXD3	3′-UTR
rs1318778	2	176173103	1	1	C	G	HOXD3	
rs1549334	2	176174469	1	1	G	A	HOXD3	
rs6433571	2	176174850	0.98	1	G	T	HOXD3	
rs2072590	2	176177905	1	1	A	C	AC009336.24	intronic
rs6755766	2	176178477	0.96	1	T	C	AC009336.24	intronic
rs6755777	2	176178498	0.99	1	T	G	AC009336.24	intronic
rs1562315	2	176180754	0.98	1	T	A	HOXD-AS1	intronic

AFR, African samples; AMR, Ad Mixed American samples; ASN, East Asian samples; EUR, European samples; LD, linkage disequilibrium; SNP, single nucleotide polymorphism; Ref = reference allele; Alt = altered allele.

### Functional annotation using RegulomeDB

RegulomeDB was used to annotate these 19 genetic variants with known and predicted regulatory elements. The results showed that three genetic variants including rs1562315, rs2551802 and rs6433571 likely to affect TF binding + any motif + DNase Footprint + DNase peak, as described in Table 2. Other genetic variants are likely to affect TF binding + DNase peak. More detailed results are described in Table 2.

**Table 2:** Functional annotation results using RegulomeDB

SNP	chromosome	pos (hg38)	Ref	Alt	Regulome DB Score
rs1562315	2	176180754	T	A	2b
rs2551802	2	176157430	C	G	2b
rs6433571	2	176174850	G	T	2b
rs1051929	2	176172026	T	C	4
rs1318778	2	176173103	C	G	4
rs1549334	2	176174469	G	A	4
rs2072590	2	176177905	A	C	4
rs2249131	2	176167367	C	T	4
rs2252894	2	176159194	G	C	4
rs2252895	2	176159192	A	G	4
rs2857538	2	176159533	C	T	4
rs2857540	2	176161970	G	T	4
rs6755766	2	176178477	T	C	4
rs6755777	2	176178498	T	G	4
rs711830	2	176172583	A	G	4
rs2113559	2	176166371	A	G	5
rs2857532	2	176168555	A	G	5
rs4972504	2	176153998	T	C	5
rs717852	2	176166895	C	T	5

1a, eQTL + TF binding + matched TF motif + matched DNase Footprint + DNase peak; 1b, eQTL + TF binding + any motif + DNase Footprint + DNase peak; 1c, eQTL + TF binding + matched TF motif + DNase peak; 1d, eQTL + TF binding + any motif + DNase peak; 1e, eQTL + TF binding + matched TF motif; 1f, eQTL + TF binding / DNase peak; 2a, TF binding + matched TF motif + matched DNase Footprint + DNase peak; 2b, TF binding + any motif + DNase Footprint + DNase peak; 2c, TF binding + matched TF motif + DNase peak; 3a, TF binding + any motif + DNase peak; 3b, TF binding + matched TF motif; 4, TF binding + DNase peak; 5, TF binding or DNase peak; 6, other.

### Functional annotation using PhenoScanner

Using PhenoScanner (version 1.1), we identified that these 19 genetic variants could significantly regulate the expression of nearby genes including HOXD-AS1, HOXD3, HOXD1, HOXD4, ATP5G3, HOXD9, HOXD11, KIAA1715, MTX2, LINC01116, HOXD-AS2, HOXD8, and HOXD10 in 32 human tissues. These tissues include Adipose subcutaneous, Adipose visceral omentum, Artery tibial, Brain cerebellar hemisphere, Brain hippocampus, Brain nucleus accumbens basal ganglia, Brain putamen basal ganglia, Breast mammary tissue, Cells transformed fibroblasts, Colon sigmoid, Colon transverse, Esophagus gastroesophageal junction, Esophagus mucosa. Esophagus muscularis, Heart atrial appendage, Lung, Lymphoblastoid cell lines, Muscle skeletal, Nerve tibial, Ovary, Pancreas, Peripheral blood, Skin, Skin not sun exposed suprapubic, Skin sun exposed lower leg, Small intestine terminal ileum, Spleen, Stomach, Testis, Thyroid, Uterus and Whole blood. Interestingly, these genetic variants could significantly regulate the gene expression of HOXD1 and HOXD3 in human ovary tissue, as described in Table 3. More detailed results in 32 human tissues are described in Supplementary Table 1.

**Table 3:** 19 genetic variants and gene expression in human ovary tissue

SNP	Pos (hg19)	Alleles	Tissue	Gene	Ensembl	*N*	Effect Allele	Beta	SE	*P*
rs1051929	chr2:177036754	T/C	Ovary	HOXD1	ENSG00000128645.11	85	T	0.4891	0.1384	0.000765
rs1051929	chr2:177036754	T/C	Ovary	HOXD3	ENSG00000128652.7	85	T	0.5701	0.1525	0.000396
rs1318778	chr2:177037831	C/G	Ovary	HOXD1	ENSG00000128645.11	85	C	0.4921	0.1367	0.000623
rs1318778	chr2:177037831	C/G	Ovary	HOXD3	ENSG00000128652.7	85	C	0.5721	0.1507	0.000328
rs1549334	chr2:177039197	G/A	Ovary	HOXD1	ENSG00000128645.11	85	G	0.4921	0.1367	0.000623
rs1549334	chr2:177039197	G/A	Ovary	HOXD3	ENSG00000128652.7	85	G	0.5721	0.1507	0.000328
rs1562315	chr2:177045482	T/A	Ovary	HOXD1	ENSG00000128645.11	85	T	0.4404	0.1334	0.00158
rs1562315	chr2:177045482	T/A	Ovary	HOXD3	ENSG00000128652.7	85	T	0.5172	0.147	0.000804
rs2072590	chr2:177042633	C/A	Ovary	HOXD1	ENSG00000128645.11	85	C	-0.4923	0.1367	0.00062
rs2072590	chr2:177042633	C/A	Ovary	HOXD3	ENSG00000128652.7	85	C	-0.5724	0.1507	0.000327
rs2113559	chr2:177031099	G/A	Ovary	HOXD1	ENSG00000128645.11	85	G	-0.4623	0.1444	0.002132
rs2113559	chr2:177031099	G/A	Ovary	HOXD3	ENSG00000128652.7	85	G	-0.5461	0.159	0.001047
rs2249131	chr2:177032095	C/T	Ovary	HOXD1	ENSG00000128645.11	85	C	0.4629	0.1441	0.002068
rs2249131	chr2:177032095	C/T	Ovary	HOXD3	ENSG00000128652.7	85	C	0.5464	0.1587	0.001019
rs2252894	chr2:177023922	C/G	Ovary	HOXD1	ENSG00000128645.11	85	C	-0.3923	0.1462	0.009254
rs2252895	chr2:177023920	A/G	Ovary	HOXD1	ENSG00000128645.11	85	A	0.4738	0.1439	0.001618
rs2252895	chr2:177023920	A/G	Ovary	HOXD3	ENSG00000128652.7	85	A	0.5187	0.1605	0.001948
rs2551802	chr2:177022158	C/G	Ovary	HOXD1	ENSG00000128645.11	85	C	0.4433	0.1374	0.001984
rs2551802	chr2:177022158	C/G	Ovary	HOXD3	ENSG00000128652.7	85	C	0.5068	0.1522	0.001447
rs2857532	chr2:177033283	A/G	Ovary	HOXD1	ENSG00000128645.11	85	A	0.4629	0.1441	0.002068
rs2857532	chr2:177033283	A/G	Ovary	HOXD3	ENSG00000128652.7	85	A	0.5464	0.1587	0.001019
rs2857538	chr2:177024261	C/T	Ovary	HOXD1	ENSG00000128645.11	85	C	0.4715	0.1435	0.001652
rs2857538	chr2:177024261	C/T	Ovary	HOXD3	ENSG00000128652.7	85	C	0.532	0.1593	0.001399
rs2857540	chr2:177026698	G/T	Ovary	HOXD1	ENSG00000128645.11	85	G	0.4197	0.15	0.006799
rs2857540	chr2:177026698	G/T	Ovary	HOXD3	ENSG00000128652.7	85	G	0.5023	0.1653	0.003439
rs4972504	chr2:177018726	C/T	Ovary	HOXD1	ENSG00000128645.11	85	C	-0.4658	0.1416	0.001635
rs4972504	chr2:177018726	C/T	Ovary	HOXD3	ENSG00000128652.7	85	C	-0.5097	0.158	0.001977
rs6433571	chr2:177039578	G/T	Ovary	HOXD1	ENSG00000128645.11	85	G	0.4584	0.1353	0.00121
rs6433571	chr2:177039578	G/T	Ovary	HOXD3	ENSG00000128652.7	85	G	0.5254	0.1497	0.000827
rs6755766	chr2:177043205	C/T	Ovary	HOXD1	ENSG00000128645.11	85	C	-0.4481	0.1368	0.001702
rs6755766	chr2:177043205	C/T	Ovary	HOXD3	ENSG00000128652.7	85	C	-0.5348	0.1502	0.000705
rs6755777	chr2:177043226	G/T	Ovary	HOXD1	ENSG00000128645.11	85	G	-0.4405	0.1334	0.001577
rs6755777	chr2:177043226	G/T	Ovary	HOXD3	ENSG00000128652.7	85	G	-0.5171	0.147	0.000807
rs711830	chr2:177037311	G/A	Ovary	HOXD1	ENSG00000128645.11	85	G	-0.4921	0.1367	0.000623
rs711830	chr2:177037311	G/A	Ovary	HOXD3	ENSG00000128652.7	85	G	-0.5721	0.1507	0.000328
rs717852	chr2:177031623	T/C	Ovary	HOXD1	ENSG00000128645.11	85	T	-0.4629	0.1441	0.002068
rs717852	chr2:177031623	T/C	Ovary	HOXD3	ENSG00000128652.7	85	T	-0.5464	0.1587	0.001019

## DISCUSSION

Overall, the GWAS methods have reported new variants that are associated with OC risk [1]. However, the exact genetic mechanisms for these OC susceptibility variants are still unclear [2]. Evidence shows that the potential associations between gene expression and OC risk alleles may connect risk variants to their putative target genes/transcripts and biological pathways [2]. Zhao et al. selected seven OC risk variants including rs3814113 on 9p22, rs2072590 on 2q31, rs2665390 on 3q25, rs10088218, rs1516982, rs10098821 on 8q24, and rs2363956 on 19p13 [2]. They evaluated the associations between gene expression and OC risk alleles using the whole genome mRNA expression data in 121 lymphoblastoid cell lines from 74 non-related familial ovarian cancer patients, and 47 non-cancer unrelated family controls [2]. They identified two cis-associations between *rs10098821* and *c-Myc*, and *rs2072590* and *HS.565379.*

The OC risk-associated SNP rs2072590 lies in non-coding DNA downstream of *HOXD3* and upstream of *HOXD1*, and it tags SNPs in the *HOXD3* 3′ UTR [3]. *However,* Zhao et al. did not report any significant association between rs2072590 and *HOXD1 or HOXD3.* We think that the non-coding rs2072590 variant may contribute to OC susceptibility by regulating the gene expression of *HOXD1 and HOXD3.* Here, we conducted a functional annotation of rs2072590 variant using RegulomeDB (version 1.1) [4], HaploReg (version 4.1) [5], and PhenoScanner (version 1.1) [6].

Using HaploReg, we identified 19 genetic variants tagged by rs2072590 variant with with r^2^ >= 0.8. Using RegulomeDB, we identified that three genetic variants are likely to affect TF binding + any motif + DNase Footprint + DNase peak. Other genetic variants are likely to affect TF binding + DNase peak. Using PhenoScanner (version 1.1), we identified that these 19 genetic variants could significantly regulate the expression of nearby genes, especially the HOXD1 and HOXD3 in human ovary tissue.

In addition to the OC, some other comprehensive functional annotation of human complex diseases have also been conducted including colorectal cancer [7, 8], prostate cancer [9–11], breast cancer [12], multiple sclerosis [13], and Alzheimer’s disease [14]. Collectively, we think that our results provide further insight into the genetic architecture of inherited susceptibility to OC, as did in previous studies [7–14].

## MATERIALS AND METHODS

### LD analysis using HaploReg

HaploReg is a tool for exploring annotations of the noncoding genome at variants on haplotype blocks [5]. HaploReg includes LD information from the 1000 Genomes Project, chromatin state and protein binding annotation from the Roadmap Epigenomics and the Encyclopedia of DNA Elements (ENCODE) projects, sequence conservation across mammals, the effect of SNPs on regulatory motifs, and the effect of SNPs on gene expression from eQTL studies [5]. We used HaploReg (version 4.1) to identify the rs2072590 tagged variants using the LD information from the 1000 Genomes Project (*EUR*) with r^2^ > = 0.8 [5].

### Functional annotation using RegulomeDB

RegulomeDB (version 1.1) is a database that annotates SNPs with known and predicted regulatory elements in the intergenic regions of the human genome [4]. Known and predicted regulatory DNA elements include regions of DNAase hypersensitivity, binding sites of transcription factors, and promoter regions that have been biochemically characterized to regulation transcription [4]. RegulomeDB (version 1.1) includes the public datasets from Gene Expression Omnibus (GEO), the ENCODE project, and published literature [4].

### Functional annotation using PhenoScanner

PhenoScanner (version 1.1) is a curated database holding publicly available results from large-scale GWAS [6]. The motivation for creating this tool is to facilitate “phenome scans”, the cross-referencing of genetic variants with a broad range of phenotypes, to help aid the understanding of disease pathways and biology [6]. The catalogue currently contains nearly 3 billion associations and over 10 million unique SNPs [6]. The results are aligned across traits to the same effect and non-effect alleles for each SNP [6].

## SUPPLEMENTARY MATERIALS TABLE




