# Supplementary material for: Ovarian cancer variant rs2072590 is associated with HOXD1 and HOXD3 gene expression
Source: Oncotarget. 2017 Oct 13;8(61):103410–4. doi: 10.18632/oncotarget.21902 (PMC5732737; doi:10.18632/oncotarget.21902)
Supplement: Supplementary file 1 [file oncotarget-08-103410-s001.pdf]

Ovarian cancer variant rs2072590 is associated with HOXD1 and HOXD3 gene expression

SUPPLEMENTARY MATERIALS

Supplementary Table 1: Detailed results from functional annotation using PhenoScanner in 32 human tissues. See Supplementary_Table 1
